# Actinomycetes: Role in Biotechnology and Medicine

**DOI:** 10.1155/2013/687190

**Published:** 2013-05-30

**Authors:** Neelu Nawani, Bertrand Aigle, Abul Mandal, Manish Bodas, Sofiane Ghorbel, Divya Prakash

**Affiliations:** ^1^Dr. D. Y. Patil Biotechnology and Bioinformatics Institute, Dr. D. Y. Patil Vidyapeeth, Pune 411033, India; ^2^Université de Lorraine, UMR 1128, Dynamique des Génomes et Adaptation Microbienne, 54506 Vandœuvre-lès-Nancy, France; ^3^INRA, Dynamique des Génomes et Adaptation Microbienne, UMR 1128, 54506 Vandœuvre-lès-Nancy, France; ^4^School of Life Sciences, System Biology Research Center, University of Skövde, Box 408, 541-28 Skövde, Sweden; ^5^Laboratoire de Génie Enzymatique et de Microbiologie, Ecole Nationale D'Ingénieurs de Sfax, Sfax, Tunisia

Actinomycetes, one of the most diverse groups of filamentous bacteria, are well recognized for their metabolic versatility. The bioactive potential of these bacteria facilitates their survival even in distress and unfavourable ecological conditions. This special issue is dedicated to the importance of multitude of primary and secondary metabolites produced by actinomycetes. The six articles published in this issue balance the biocatalytic and biocidal potential of actinomycetes.

The importance of large repertory of enzymes from actinomycetes and their potential in replacing chemical catalysts is discussed. Successful commercialization of these enzymes is an important step towards revolutionizing “green technology.” Reduction in the cost of enzyme production is demonstrated by production of endoglucanases from *Streptomyces* sp. on low-cost substrates. Such low-cost production initiatives can be extended to other enzymes and metabolites. Novel properties like thermal and ionic stabilities and a better turnover make these systems infallible and regenerative. The activity of enzymes from actinomycetes is not confined to substrate conversion alone but broadened to biocontrol of quorum-sensing-dependent phytopathogens, as mediated by acyl-homoserine-lactone-degrading enzymes from endophytic actinomycetes.

Unexplored environments often appeal to researchers in the hope of accruing novel bacteria, a continuous quest which has actually led to discovery of unusually industrious microbes. Antimicrobial potential of actinobacteria isolated from the integument of *Trachymyrmex* fungus-growing ants is on par with commercial antimicrobials, clearly manifesting a new explorable niche “actinobacterial symbionts of plants and animals.” The term “antimicrobials” often leads our thoughts to “medicine-related” but it's “environment-related” applications are less contrived. *Streptomyces lunalinharesii* produces antimicrobial substances against sulfate-reducing bacteria commonly responsible for corrosion in the petroleum industry, with an ability to replace the existing biocides. Making the best out of the already good can be achieved for actinomycetes by strain improvement. Advanced microarray-driven reverse engineering strategies for the understanding and modulation of independently functioning regulatory pathways can allow these microfactories to overproduce important antibiotics.

In a nutshell, actinomycetes offer the most promising synthesizers of many industrially and commercially meaningful metabolites. Novel and unexplored habitats may offer bacterial assemblages not reached hitherto. An integration of newer habitats, screening, and improvement technologies can offer promising candidates for biotechnology and health-related applications.

*Neelu Nawani* *Bertrand Aigle* *Abul Mandal* *Manish Bodas* *Sofiane Ghorbel* *Divya Prakash*

